# Integration of metabolomics and network pharmacology to reveal the protective mechanism underlying Qibai Pingfei capsule on chronic obstructive pulmonary disease

**DOI:** 10.3389/fphar.2023.1258138

**Published:** 2023-10-18

**Authors:** Jinghui Xie, Mengxiang Liu, Yating Gao, Changan Liu, Fan Wu, Jiabing Tong, Zegeng Li, Jie Zhu

**Affiliations:** ^1^ College of Integrated Chinese and Western Medicine, Anhui University of Chinese Medicine, Hefei, Anhui, China; ^2^ Center for Xin’an Medicine and Modernization of Traditional Chinese Medicine, Institute of Health and Medicine, Anhui University of Chinese Medicine, Hefei, China; ^3^ The First Affiliated Hospital of Anhui University of Traditional Chinese Medicine, Hefei, Anhui, China; ^4^ College of Traditional Chinese Medicine, Anhui University of Chinese Medicine, Hefei, Anhui, China; ^5^ Institutes of Integrative Medicine, Fudan University, Shanghai, China

**Keywords:** COPD, Qibai Pingfei capsule, metabolomics, network pharmacology, ferroptosis, glutathione metabolism

## Abstract

In this study, we have employed metabolomics technology in combination with network pharmacology to ascertain the key metabolites and hub genes. The objective was to explore the pathway of Qibai Pingfei Capsule (QBPF) in treating COPD through metabolomics. We identified 96 differential metabolites in the lung tissues of rats belonging to control and model groups, out of which 47 were observed to be critical (VIP >2, *p* < 0.05). Furthermore, 16 important differential metabolites were reversed after QBPF treatment. Using network pharmacology, we identified 176 core targets of 81 drug-active ingredients. Our comprehensive analysis of network pharmacology and metabolomics enabled us to identify a core target, prostaglandin-endoperoxide synthase 2 (PTGS2), and a core metabolic pathway for glutathione metabolism. Finally, the result of molecular docking showed that PTGS2 had strong binding activity to 18 compounds including Fumarine and Kaempferol, etc.. PTGS2 is a marker of ferroptosis, so we wanted to explore whether QBPF could inhibit ferroptosis in COPD. The results showed that ferroptosis was involved in the pathogenesis of COPD, and QBPF could inhibit the occurrence of ferroptosis. In conclusion, the mechanism of QBPF for treating COPD may be related to PTGS2 expression, glutathione metabolism and ferroptosis.

## 1 Introduction

As a disease with high morbidity and mortality worldwide, COPD attracts the attention of traditional and clinical medicine. COPD is currently one of the top three causes of death worldwide, with 90% of deaths occurring in low- and middle-income countries (Meghji et al., 2021). In recent years, studies attempted to discover biomarkers for COPD diagnosis in urine, plasma, serum, and sputum samples (Adamko et al., 2015; Esther et al., 2022; Paris et al., 2022). With the development and progress of omics technology, single-cell sequencing technology and metabolomics technology can analyze the occurrence and development of COPD, and facilitate the diagnosis of COPD. Metabolomics emerged in the 1990s to identify and analyze complex metabolites (Alsaleh et al., 2019). COPD-related metabolomics-driven biomarkers can elucidate the pathophysiological mechanisms, with the ultimate goal of providing more accurate diagnosis and personalized treatment from an informed perspective. Early studies have shown that patients with COPD experience metabolic dysregulation. For example, Kalle et al. showed that lipid metabolism abnormalities, particularly sphingolipid metabolism disorders, can occur in patients with COPD (Kilk et al., 2018). In addition, Zhou and his team explored COPD biomarkers at different stages using plasma metabolomics and lipid metabolomics (Zhou et al., 2020). It can be seen that there are abnormal changes in the metabolic level of COPD. Therefore, it is necessary to study the occurrence and development of COPD from the metabolic perspective.

At present, there is no specific therapy available for the treatment of COPD. Palliative therapy remains the mainstream approach to managing this incurable disease, with the aim of improving airway limitation (Zhou et al., 2020). Traditional Chinese medicine was demonstrated proven effectiveness in improving various clinical symptoms of COPD and reducing the use of antibiotics, as evidenced in prior research. QBPF is a traditional Chinese medicine compound preparation for the treatment of COPD created under the guidance of Xin’an Medicine. It is an in-hospital traditional Chinese medicine compound preparation of the First Affiliated Hospital of Anhui University of Traditional Chinese Medicine. QBPF, which are composed of traditional Chinese medicines like astragalus, Allium macrostemon Bunge, ginseng, Ligusticum chuanxiong Hort, Lepidium seed, schisandra, and earthworm, have been found to improve lung function and alleviate symptoms in COPD rats (Zhu et al., 2017). The capsule has the effect of replenishing qi, resolving phlegm and removing blood stasis. With the more than 20 years of clinical treatment, it has been proven that QBPF could effectively improve the lung function of patient and have a certain improvement effect on lung inflammation. In addition, animal studies also confirmed that QBPF could improve the lung function of COPD rats by regulating the microecology of the lung-gut axis and downregulate the expression of inflammatory factors such as IL-10 and IL-17A, etc. (Jia et al., 2022). Furthermore, our experimental study indicated that QBPF exhibited good stability through HPLC fingerprint analysis and was approved as a new drug (The patent number is ZL 2010 10573274.1) (FU Juan et al., 2017). However, further research is needed to elucidate the specific therapeutic mechanism of QBPF on COPD.

Traditional Chinese medicine (TCM) formulations possess the ability to impact the entire body due to their diverse composition and multi-target effects. The utilization of metabolomics technology is highly beneficial in detecting the dynamic changes of metabolites following herbal medicine treatment. However, when metabolomics is employed as an independent research methodology, it cannot fully explain the therapeutic mechanism of TCM prescriptions in treating diseases. In this regard, network pharmacology is a suitable solution to supplement metabolomics’ inadequacies. By assessing the pharmacological effects of multiple drug targets in compound Chinese medications at the molecular level, network pharmacology effectively compensates for the limitations of metabolomics. Therefore, combining metabolomics and network pharmacology to predict compound-target combinations and perform related pathway analysis enables a better understanding of the endogenous metabolic-level therapeutic effect of TCM compounds on diseases.

**FIGURE 1:**
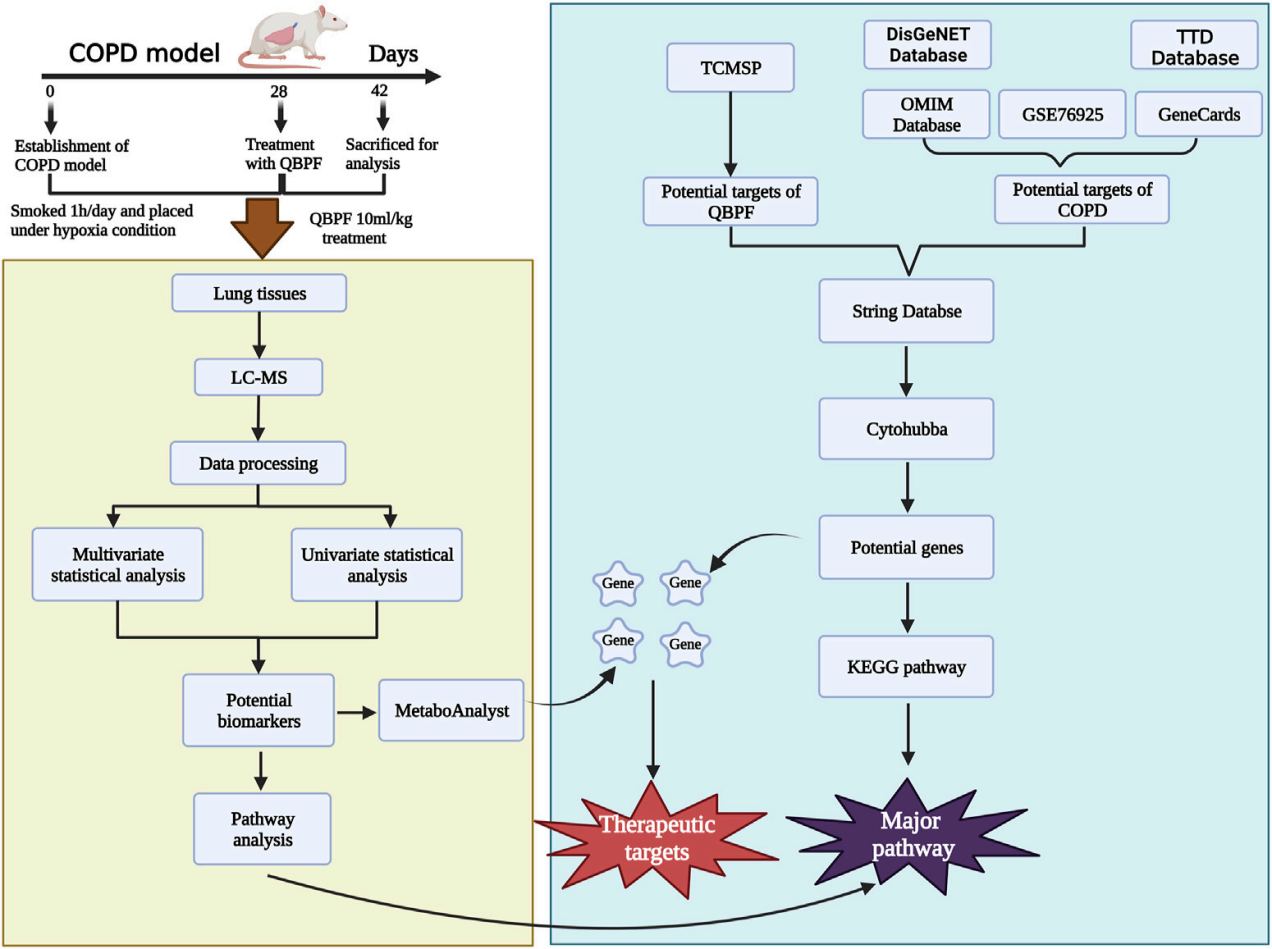
Workflflow of metabolomics and network pharmacology analysis.

Therefore, the purpose of this study was to comprehensively explore the effective approach of QBPF in treating COPD through integrating metabolomics technology and network pharmacology. Initially, network pharmacology was used to predict the effective targets of QBPF for treating COPD, followed by utilizing metabolomics techniques to identify metabolites and metabolic pathways. Eventually, the shared objective of both technologies was examined to scrutinize the therapeutic mechanism of QBPF further. This study contributes to establishing the correlation between key biomarkers and core genes and provides a theoretical research basis for the QBPF treatment of COPD. We put on the flow chart of the research process is displayed in Figure 1.

## 2 Materials and methods

### 2.1 Animals

Twenty-four Specific pathogen-free (SPF)-grade Sprague–Dawley (SD) strain of rats with a weight of (200 ± 20) g were purchased from the Huaxing Experimental Animal Farm, Huiji District, Zhengzhou City (SCXK 2019-0002; Zhengzhou, China). They were divided into three groups, including control group (CON), model group (COM) and QBPF capsule treatment group (QBPF). All experiments were performed following the Experimental Animal Ethics Committee of the Anhui University of Chinese Medicine (identification number: AHUCM-rats-2022023).

### 2.2 Construction of COPD model and obtention of rats sample

The COPD model was established for all rats except the normal group: the rats were placed in a self-made plexiglass smoke box. Xiongshi brand cigarettes (Tar content: 13 mg, nicotine content: 0.9 mg, carbon monoxide content: 14 mg, China Tobacco Zhejiang Industrial Co., Ltd.) were inserted into the cigarette lighting box, the micro-vacuum pump was turned on after lighting to suck the smoke into the smoking box through the negative pressure. Twenty cigarettes were used each time, lasting for 1 h. After smoking, the rats were placed in a normal-pressure hypoxic device (6 h a day), and fed with nitrogen, and the oxygen concentration was adjusted to 10% by using an automatic oxygen meter. The CO_2_ concentration in the control cabin was always maintained at 0.03%. The sensor and its control circuit constantly kept the temperature in the cabin at 22°C-24°. The whole cabin is made of transparent plexiglass, which allows for monitoring rats’ eating, activities, and drinking water. Modeling was performed 6 days a week, suspended on the seventh day, for 4 consecutive weeks. Two weeks after the modeling, the QBPF-treated group was administered 10 mL/kg per day (50 mg of QBPF capsule per milliliter), and the rats in the control group and the model group were orally given an equal volume of sterile normal saline, once a day.

2% pentobarbital sodium (1 mL/kg) was intraperitoneally injected to anesthetize the rats. After obtaining the pulmonary function data, the abdominal cavity and thorax of the rat were cut open with surgical forceps, blood was collected from the abdominal aorta and lung tissue was cut, and then taken for further testing.

### 2.3 Pulmonary function test

Using the animal lung function analysis system AniRes 2005 (Beijing Belambo Technology Co., Ltd.): 2% pentobarbital sodium (1 mL/kg) was intraperitoneally injected to anesthetize the rats. Animals were fixed on the mouse board, we trimmed their neck fur and sterilized the area. We longitudinally incised the skin of their neck, and separated the subcutaneous tissue and sternohyoid muscle to expose the trachea. Using the tissue scissors, we horizontally cut the trachea between the cartilage rings of the trachea and inserted a tracheal tube. The tube was connected to a three-way switch in the middle of the trachea, and placed in the supine position. Forced expiratory volume in 0.3 s (FEV0.3), forced expiratory vital capacity (FVC), forced expiratory volume in the first 0.3 s, the percentage of forced expiratory vital capacity (FEV0.3/FVC)%, and other parameters of pulmonary function test were measured.

### 2.4 Pathological observation of lung tissue

The upper lobe of the right lung was separated and treated with 0.9% sodium chloride solution After rinsing, the surface moisture was blotted with filter paper, and then fixed in 4% formaldehyde for 48 h. Afterwards, paraffin embedding, sectioning, and slide preparation were carried out, and finally hematoxylin-eosin staining (HE) was performed to observe the pathological form of lung tissue.

### 2.5 Transmission electron microscopy

1 cm^3^ of lung tissue was fixed to 2.5% Glutaraldehyde for 12 h. After rinsing with PBS, fix with 1% osmic acid for 2 h. Afterwards, dehydrating, embedding, slicing, and staining were performed. Digital images were obtained using a Transmission electron microscopy.

### 2.6 Malondialdehyde, lactate dehydrogenase, and iron assay

The lung tissue was frozen in liquid nitrogen and then ground into a powder. The contents of MDA, LDH and iron in rat lung tissue were measured with corresponding kits according to the manufacturer’s instructions.

### 2.7 Network pharmacology

The chemical constituents of QBPF were obtained from the Traditional Chinese Medicine Systems Pharmacology Database and Analysis Platform (TCMSP, https://old.tcmsp-e.com/tcmsp.php). The active ingredients of earthworm were obtained from an article (Bao et al., 2022). Subsequently, the effective chemical components were entered into the Pubchem database (https://pubchem.ncbi.nlm.nih.gov/) to obtain their structural formula. Then, the structural formula was imported into the SwissTargetPrediction (http://www.swisstargetprediction.ch/) database to obtain the corresponding target. Targets and candidate genes related to chronic obstructive pulmonary disease (COPD) were obtained from OMIM (https://omim.org/), Genecards (https://www.genecards.org/), TTD (https://idrblab.org/ttd/), and DisGeNET (https://www.disgenet.org/), using the keywords “COPD” or “chronic obstructive pulmonary disease”. The GSE76925 dataset was derived from the gene expression omnibus (GEO, https://www.ncbi.nlm.nih.gov/geo/), and COPD-related targets were obtained using screening criteria of *p* < 0.05 and |log2FC| > 0. The targets of QBPF, COPD, and GSE76925 were synthesized, intersected, and inputted into the STRING website (https://string-db.org/) to construct a protein-protein interaction (PPI) network. Hub genes were obtained using the CytoHubba plugin in Cytoscape software. Metascape (https://metascape.org/) facilitated gene ontology (GO) enrichment of potential targets and KEGG pathway (*p* < 0.05) analysis.

### 2.8 Molecular docking

Use AutoDock Vina software (http://vina.scripps.edu/) to prepare the ligands and proteins required for molecular docking. For the target protein, obtain the crystal structure in the PDB database (https://www.rcsb.org/) Preprocessing is required, including removing hydrogenation, modifying amino acids, optimizing energy and adjusting force field parameters, and satisfying the low-energy conformation of the ligand structure after downloading the ligand structure (https://pubchem.ncbi.nlm.nih.gov/). Finally, the molecular docking of the target structure and the active ingredient structure is carried out, and the internal vina of the pyrx software (https://pyrx.sourceforge.io/) is used for docking, and the Affinity (kcal/mol) value represents the combination of the two Ability, the lower the binding ability, the more stable the binding between the ligand and the receptor. It was visualized and analyzed using Pymol (https://pymol.org/2/). (The lower the binding energy, the better the binding).

### 2.9 Western blot

Add lung tissue to RIPA buffer for lysis. Collect the supernatant, which contains the total protein of the tissue. The protein sample was loaded into the sample hole of the SDS-PAGE gel, electrophoresed at a constant voltage of 80 v for about 1 h, and then transferred onto PVDF membranes. After 1 h of blocking in 5% milk, the membranes were incubated with antibodies targeting PTGS2, GPX4 or *β*-actin at 4°C overnight. Then the membrane was incubated with HRP-conjugated secondary antibody for 1.2 h at room temperature. ECL luminescence kit was used to detect proteins, and ImageJ software was used to analyze film bands.

### 2.10 Quantitative real-time PCR

Fifty milligrams of lung tissue were weighed and subsequently cut and ground into a fine powder using liquid nitrogen. The powder was then collected, and 1 mL TRIzol was added to facilitate cracking. Subsequently, the total RNA was extracted and reverse-transcribed into cDNA. The relative expression levels of target genes were determined by the 2^−ΔΔCT^ method. The sequence of primers used in the study is provided in Table 1.

**TABLE 1:** The primers used in this study.

Gene	Forward primer	Reverse primer
GPX4	AAT​CCT​GGC​CTT​CCC​TTG​CA	GCC​CTT​GGG​CTG​GAC​TTT​CA
PTGS2	TGA​TCG​AAG​ACT​ACG​TGC​AA	CTG​ATA​CTG​GAA​CTG​CTG​GT

### 2.11 Tissue collection and processing in metabolomic

80 mg of lung tissues were weighted, 20 uL each of internal standard (L-2-chlorophenylalanine, 0.3 mg/mL; Lyso PC17:0, 0.01 mg/mL, both in methanol) and 1 mL of methanol:water (V:V = 7:3) were added. Two small steel beads were added, tissue was placed at −20°C for 2 min to pre-cool, and added to the grinder (60 Hz, 2 min).Then, tissue was extracted with ultrasound for 30 min, leaved at −20°C for 20 min and centrifuged for 10 min (13,000 rpm, 4°C). 300 μL of supernatant was evaporated. Then re-dissolved with 400 μL of methanol-water (V:V = 1:4), eddied for 30 s, sonicated for 2 min, and centrifuged for 10 min (13,000 rpm, 4°C). Eventually, 150 μL of supernatant was aspirated by a syringe of supernatant, filterred by a 0.22 μm organic phase pinhole filter, and stored at −80°C with LC injection vials. When LC- MS analysis was performed, quality control samples (QC) were prepared by mixing equal volumes of extracts from all samples, and each volume of QC was as same as the volume of the sample.

### 2.12 Column conditions and mass spectrometry conditions

Chromatographic column: ACQUITY UPLCBEHC18 (100 mm × 2.1 mm, 1.7 um); column temperature: 45°C; mobile phase: A-water (containing 0.1% formic acid), B-acetonitrile/methanol (2/3) (v/v) (containing 0.1% formic acid); flow rate: 0.4 mL/min; injection volume: 5 uL.

Ion source: ESI; sample mass spectrometry signal acquisition was performed in positive and negative ion scan mode, respectively.

### 2.13 Data preprocessing and statistical analysis

The original LC-MS data was processed by software Progenesis QI V2.3 (Nonlinear, Dynamics, Newcastle, United Kingdom) for baseline filtering, peak identification, integral, retention time correction, peak alignment, and normalization. Main parameters of 5 ppm precursor tolerance, 10 ppm product tolerance, and 5% product ion threshold were applied. Compounds identification were based on precise mass-to-charge ratio (m/z), secondary fragments, and isotopic distribution using the Human Metabolome Database (HMDB), Lipidmaps (V2.3), Metlin, EMDB, PMDB, and self-built databases to make qualitative analysis.

The extracted data was then further processed by removing any peaks with a missing value (ion intensity = 0) in more than 50% in groups, by replacing zero value by half of the minimum value, and by screening according to the qualitative results of the compound. Compounds with resulting scores below 36 (out of 60) points were also deemed to be inaccurate and removed. A data matrix was combined from the positive and negative ion data.

In multivariate statistical analysis, unsupervised principal component analysis (PCA) was firstly used to observe the overall distribution among the samples and the stability of the whole analysis process. Then, supervised partial least squares analysis (PLS-DA) and orthogonal partial least squares analysis (OPLS-DA) were used to distinguish the overall differences in metabolic profiles among the groups. In order to find variables causing the differences among the groups, variable importance analysis (VIP) > 1 was set as the criterion.

Statistical analysis was performed using SPSS 16.0 data processing software. One-way ANOVA was used among multiple groups, and *t*-test was used for comparison between two groups. *P* < 0.05 was used to represent statistically significant differences.

## 3 Result

### 3.1 Metabolomics analysis

#### 3.1.1 Multivariate analysis

**FIGURE 2:**
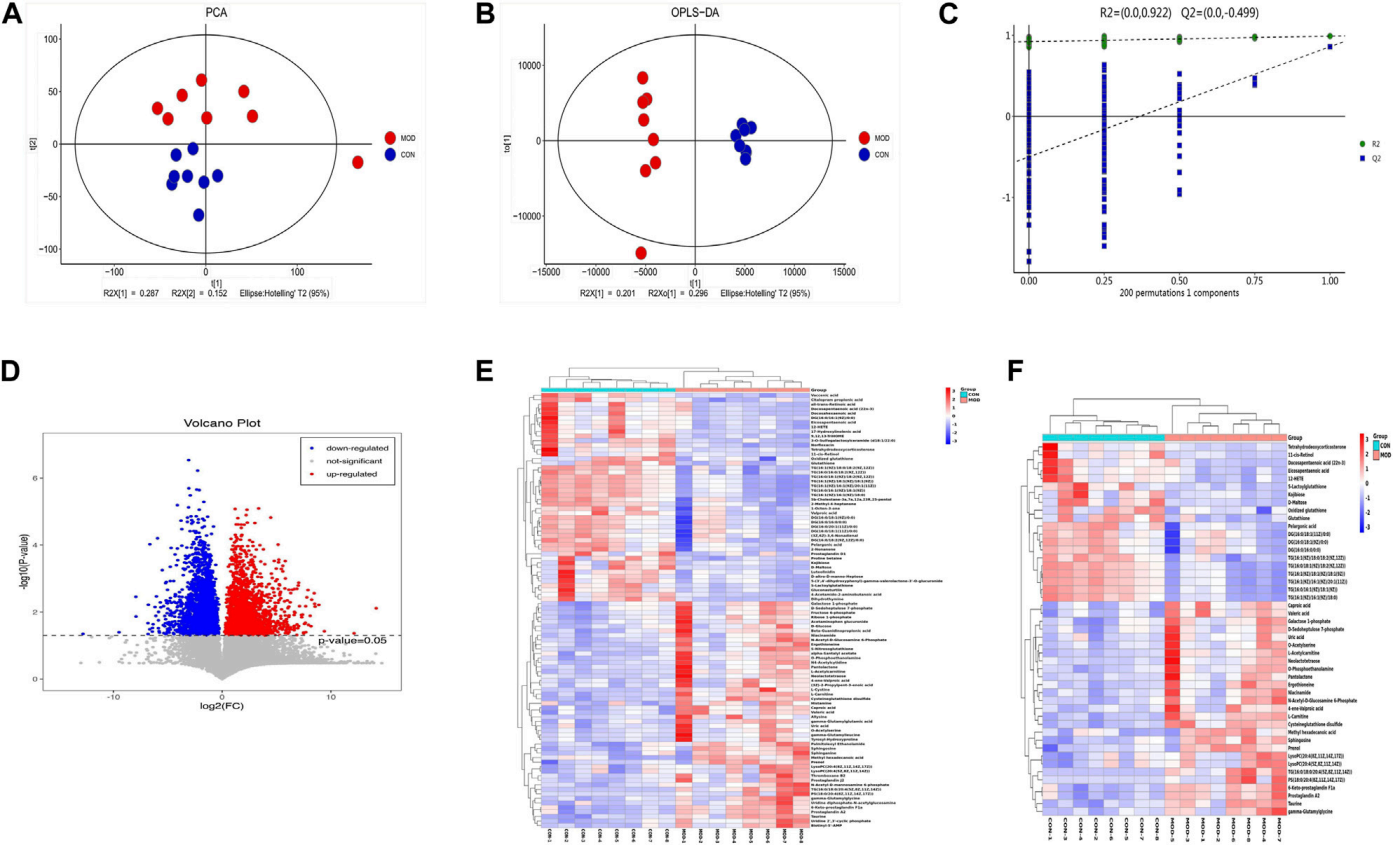
Multivariate Statistical Analysis of Metabolic Characteristics of Lung Tissue in COPD Rats. **(A)** Scores plot of PCA. **(B)** Scores plot of OPLS-DA. **(C)** Results of cross validation. **(D)** Volcano plot. **(E)** Heatmap of differential metabolites in control group and model group. **(F)** Heatmap of differential metabolites with VIP >2 compared with normal group.

Firstly, we used the PCA scatter plot to describe the discrete trend of the model group and the control group (Figure 2A). The model group and the control group were clearly different. Afterward, differential metabolites related to COPD were compared between the model group and the control group using the OPLS-DA score chart (Figure 2B). The metabolic profiles of COPD rats were significantly altered. Finally, the permutation test was used to assess the fitting degree between the data and model and the prediction effect of the model (Figure 2C). The R2X (cum), R2Y (cum), Q2 (cum), R2 intercept and Q2 intercept between the model group and the control group were 0.692, 0.991, 0.86, 0.922, and −0.499, respectively. R2 > 0.5 indicated that the fitting degree of the model is better; Q2 > 0.5 indicated that the prediction effect of the model is better, and the Q2 intercept of less than 0 indicated that the model is effective. Then, volcano plot (Figure 2D) for the normal and model groups indicated that differential metabolites were well separated. In summary, the data in all aspects of the model was valid for the next steps of screening.

Differential metabolites were screened by VIP value and *p*-value of independent sample *t*-test. In total, 96 differentially expressed metabolites were compared between the model group and the control group. Based on VIP >2 and *p* < 0.05, 47 significant differential metabolites were further screened (Table 2), which were regarded as potential differential metabolites for subsequent experiments. To better analyze these potential differential metabolites, we performed a cluster analysis on 96 differential metabolites and 47 potential biomarkers, and the heatmaps were shown in Figures 2E, F.

**TABLE 2:** The information of 47 significant differential metabolites.

Metabolites	HMDB ID	m/z	TR (min)	VIP_M_	FC	Trend
TG(16:1(9Z)/18:1(9Z)/18:1(9Z))	HMDB0005438	879.74	15.12	19.31	0.54	↓**
TG(16:1(9Z)/16:1(9Z)/20:1(11Z))	HMDB0005434	895.71	15.03	17.92	0.54	↓**
TG(16:0/18:1(9Z)/18:2(9Z,12Z))	HMDB0005384	874.78	15.12	17.45	0.51	↓**
L-Acetylcarnitine	HMDB0000201	204.12	1.14	13.89	2.19	↑**
TG(16:0/18:0/20:4(5Z,8Z,11Z,14Z))	HMDB0005370	900.80	15.23	12.89	18.38	↑**
L-Carnitine	HMDB0000062	162.11	0.76	11.05	2.35	↑**
Cysteineglutathione disulfide	HMDB0000656	427.09	0.87	8.75	2.93	↑**
Galactose 1-phosphate	HMDB0000645	259.02	0.74	7.00	1.50	↑**
TG(16:0/16:1(9Z)/18:1(9Z))	HMDB0005377	853.72	15.12	6.89	0.53	↓**
Eicosapentaenoic acid	HMDB0001999	303.23	11.39	6.57	0.55	↓**
Niacinamide	HMDB0001406	123.06	1.14	5.88	1.47	↑*
Taurine	HMDB0000251	126.02	0.71	5.61	1.46	↑**
TG(16:1(9Z)/18:0/18:2(9Z,12Z))	HMDB0005425	879.74	0.54	5.33	0.57	↓**
6-Keto-prostaglandin F1a	HMDB0002886	353.23	6.62	5.10	1.68	↑**
LysoPC(20:4(8Z,11Z,14Z,17Z))	HMDB0010396	544.34	10.82	4.84	1.46	↑*
TG(16:1(9Z)/16:1(9Z)/18:0)	HMDB0005430	848.77	15.20	4.59	0.43	↓**
Oxidized glutathione	HMDB0003337	611.14	1.16	4.45	0.68	↓*
Kojibiose	HMDB0011742	365.11	0.81	4.31	0.35	↓*
12-HETE	HMDB0006111	319.23	11.39	4.10	0.52	↓**
Glutathione	HMDB0000125	308.09	1.14	3.87	0.50	↓**
DG(16:0/18:1(9Z)/0:0)	HMDB0007102	577.52	0.44	3.62	0.89	↓**
PS(18:0/20:4(8Z,11Z,14Z,17Z))	HMDB0010165	812.54	12.51	3.43	48.68	↑*
Uric acid	HMDB0000289	169.04	1.14	3.13	1.81	↑*
D-Maltose	HMDB0000163	387.11	0.89	2.91	0.26	↓**
Sphingosine	HMDB0000252	300.29	10.02	2.84	1.66	↑**
DG(16:0/16:0/0:0)	HMDB0007098	551.50	0.40	2.81	0.92	↓*
Caproic acid	HMDB0000535	134.12	0.88	2.79	1.74	↑**
O-Phosphoethanolamine	HMDB0000224	142.03	0.69	2.77	1.99	↑**
Pelargonic acid	HMDB0000847	159.14	0.44	2.75	0.85	↓**
Tetrahydrodeoxycorticosterone	HMDB0000879	317.25	12.47	2.73	0.33	↓*
Valeric acid	HMDB0000892	120.10	0.69	2.65	1.72	↑**
O-Acetylserine	HMDB0003011	130.05	1.18	2.56	2.01	↑*
Pantolactone	HMDB0059876	146.12	0.83	2.54	1.84	↑*
Docosapentaenoic acid (22n-3)	HMDB0006528	331.26	11.90	2.43	0.37	↓*
N-Acetyl-D-Glucosamine 6-Phosphate	HMDB0001062	300.05	0.79	2.40	1.75	↑*
DG(16:0/18:1(11Z)/0:0)	HMDB0007101	577.52	15.96	2.38	0.92	↑*
S-Lactoylglutathione	HMDB0001066	360.09	4.23	2.32	0.40	↓**
Methyl hexadecanoic acid	HMDB0061859	271.26	15.96	2.32	1.10	↑*
Neolactotetraose	HMDB0006567	730.24	0.87	2.29	3.25	↑*
4-ene-Valproic acid	HMDB0013897	160.13	0.88	2.29	2.21	↑*
Ergothioneine	HMDB0003045	230.10	0.88	2.28	1.93	↑**
gamma-Glutamylglycine	HMDB0011667	205.08	0.81	2.27	1.66	↑**
Prenol	HMDB0030124	104.11	14.18	2.22	1.11	↑**
D-Sedoheptulose 7-phosphate	HMDB0001068	289.03	0.74	2.21	1.48	↑*
LysoPC(20:4(5Z,8Z,11Z,14Z))	HMDB0010395	588.33	10.83	2.06	1.42	↑*
Prostaglandin A2	HMDB0002752	335.22	6.62	2.04	1.69	↑**
11-cis-Retinol	HMDB0006216	269.23	13.15	2.00	0.18	↓*

There was difference between CON, and MOD (*p < 0.05, **p < 0.01).

#### 3.1.2 Effect of QBPF on potential biomarkers

**FIGURE 3:**
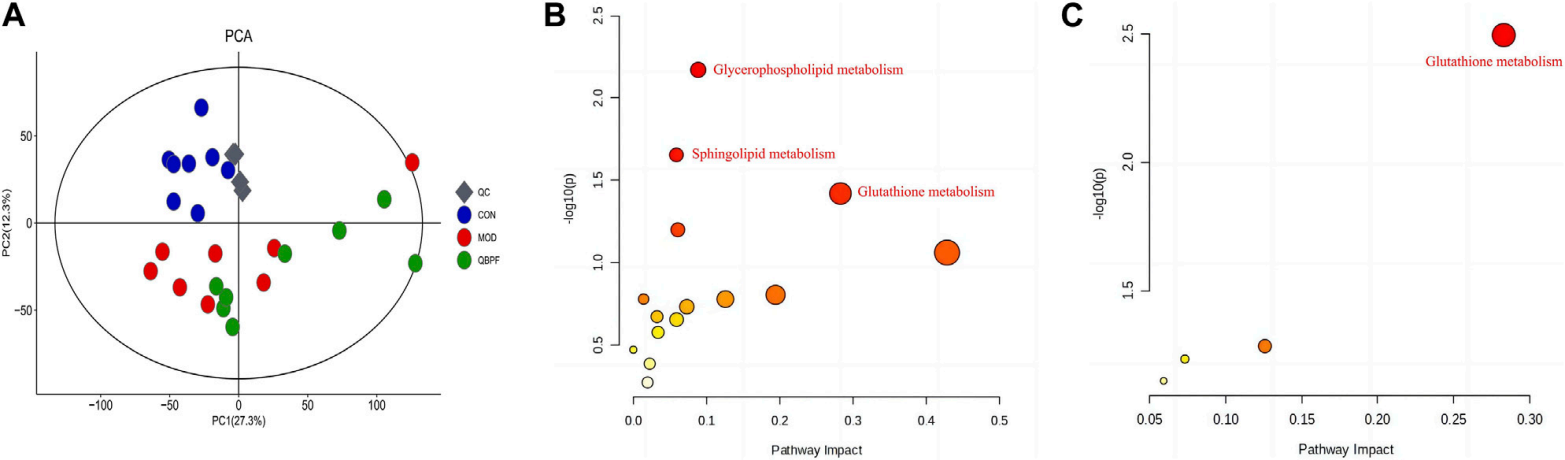
Effect of QBPF on potential biomarkers. **(A)** Scores plot of PCA among all groups. **(B)** Metabolic pathways involved in the pathogenesis of COPD. **(C)** Metabolic pathways in COPD rats under QBPF treatment. [There was difference between CON and MOD (*p < 0.05, **p < 0.01). There was difference between MOD and QBPF (^**#**^ *p* < 0.05, ^**##**^ *p* < 0.01). There was difference between CON and QBPF (^**+**^ *p* < 0.05, ^**++**^ *p* < 0.01)].

**FIGURE 4:**
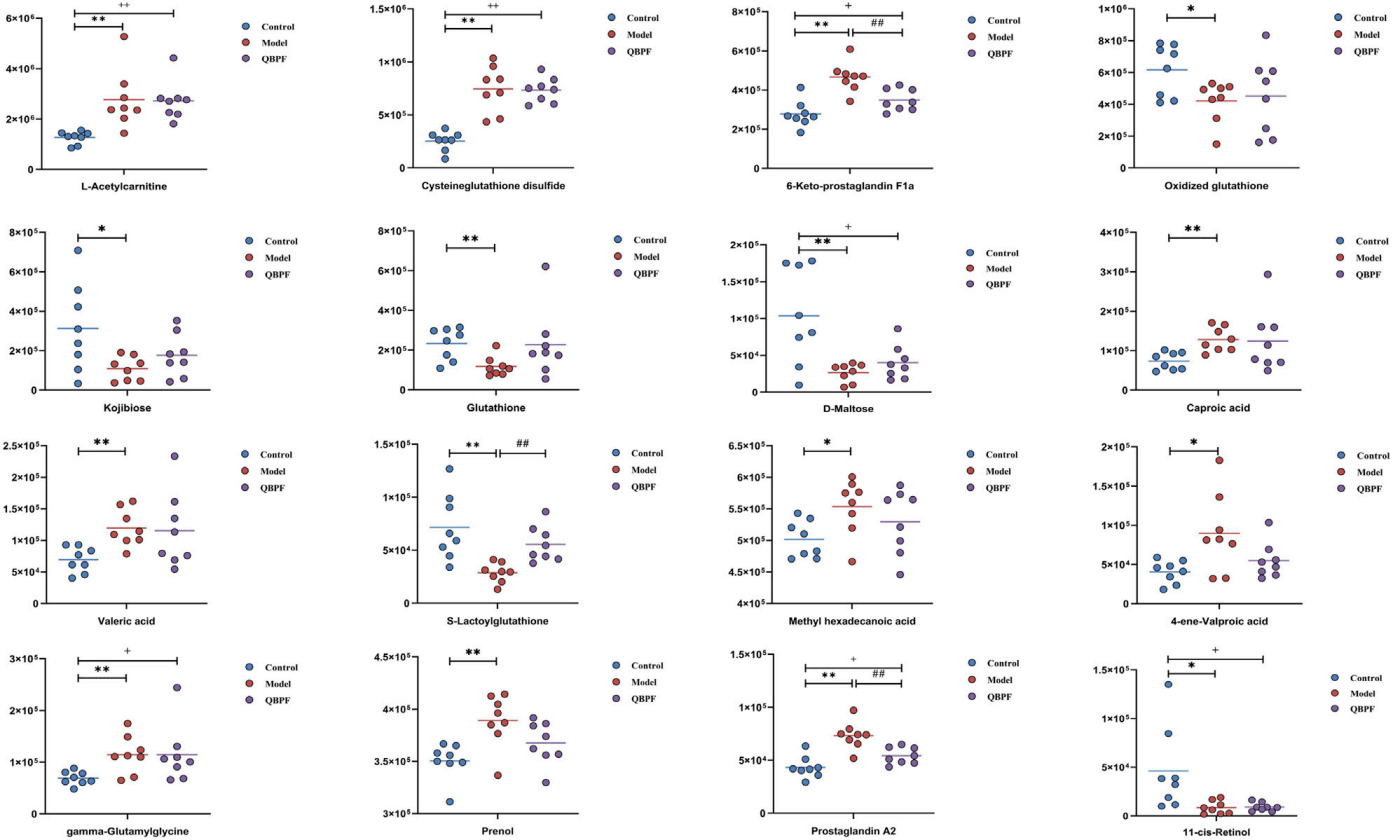
Scatterplot of the 16 potential biomarkers.

Lung tissues were collected on the 42nd day, and PCA analysis was performed (Figure 3A). Among each group, relatively obvious clusters were distributed significantly in different positions. The location of the QBPF-treated group approached of the control group, indicating that QBPF can improve the abnormal metabolic network in the rat model of COPD. Following QBPF treatment, we investigated the modulatory effect of QBPF on potential biomarkers in the rat model of COPD. Among potential 47 biomarkers, QBPF affected 16 potential biomarkers, including L-Acetylcarnitine, Cysteineglutathione disulfide, 6-Keto-prostaglandin F1a, Oxidized glutathione, Kojibiose, Glutathione, D-Maltose, Caproic acid, Valeric acid, S-Lactoylglutathione, Methyl hexadecanoic acid, 4-ene-Valproic acid, gamma-Glutamylglycine, Prenol, Prostaglandin A2 and 11-cis-Retinol. We drew a scatterplot of the 16 potential biomarkers (Figure 4).

#### 3.1.3 Metabolic pathway analysis

The metabolic pathway analysis for differential metabolites can effectively examine the impact of metabolites on diseases. The biomarkers were introduced into MetaboAnalyst 5.0 for metabolic pathway analysis, as illustrated in Figures 3B, C and Supplementary Material S1. Using a screening condition of *p* < 0.05, the significantly altered biomarkers in the model group were primarily involved in glutathione metabolism, sphingolipid metabolism, and glycerophospholipid metabolism compared to the normal group. In contrast, the callback biomarkers affected by QBPF treatment were primarily related to glutathione metabolism, suggesting that QBPF mainly treated COPD by regulating glutathione metabolism.

### 3.2 Network pharmacology

#### 3.2.1 Identification of potential targets through network pharmacology

**FIGURE 5:**
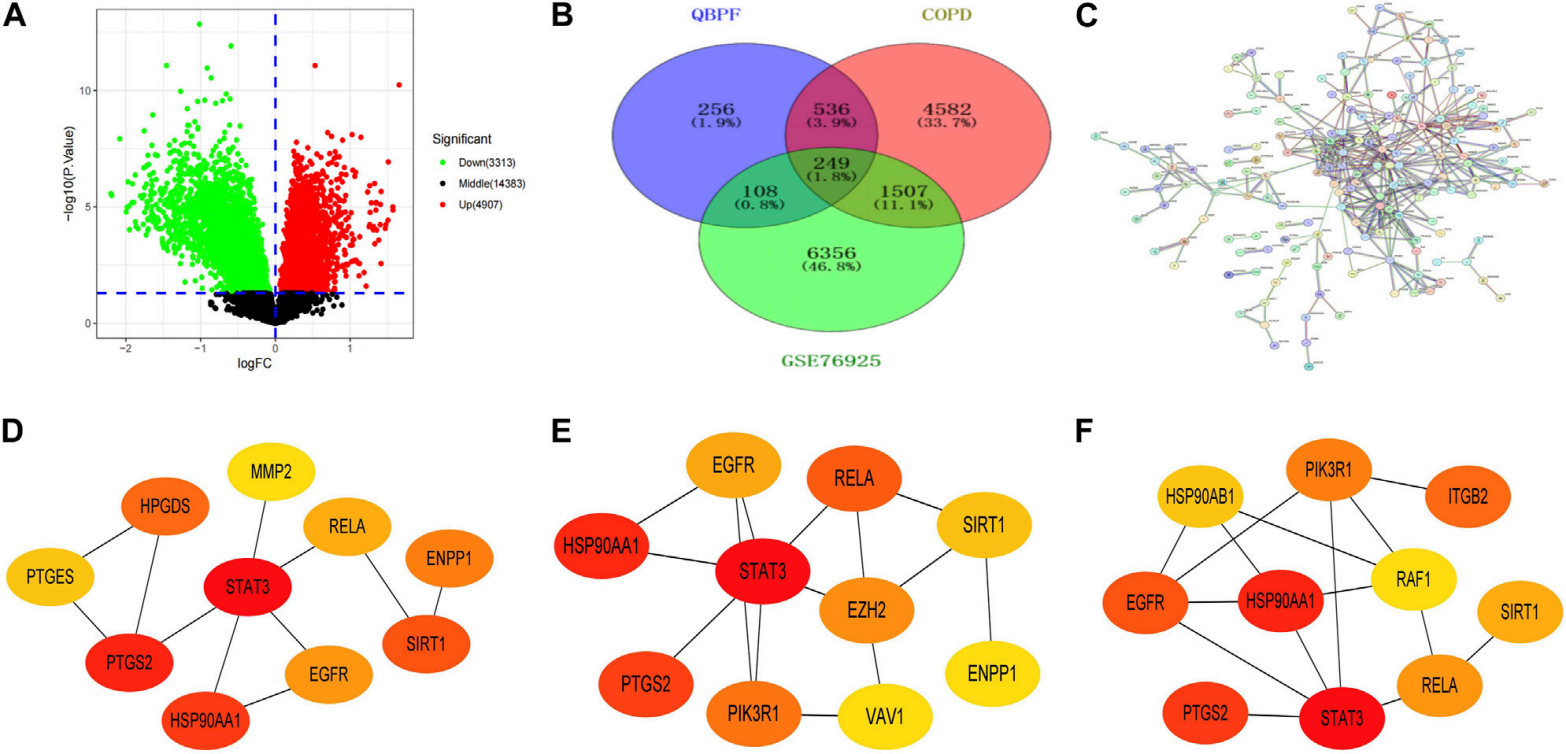
**(A)** Volcano plot of GSE76925. **(B)** Venn diagram of the intersection targets among COPD, QBPF and GSE76925. **(C)** The PPI network of QBPF treats COPD. **(D)** The top 10 genes calculated by the Betweenness algorithm in cytohubba. **(E)** The top 10 genes calculated by the BottleNeck algorithm in cytohubba. **(F)** The top 10 genes calculated by the stress algorithm in cytohubba.

We employed specific screening criteria with OB > 30% and DL > 0.18 to obtain 81 active components of QBPF from the TCMSP database and the article of earthworm. Using the Pubchem and SwissTargetPrediction databases to obtain the corresponding targets of these 81 active ingredients, 1,149 targets were obtained after deduplication. We identified 6,874 COPD candidate targets from the OMIM, Genecards, TTD, and DisGeNET databases to further refine the selection of targets for COPD treatment. To increase the precision of our target selection, we downloaded the dataset GSE76925 from the GEO database, which contains a genome-wide analysis of lung tissues of 111 COPD cases and 40 control smokers. By applying screening criteria of |log2FC| > 0 and *p* < 0.05, 8220 COPD differentially expressed genes were identified (Supplementary Material S2) (Figure 5A). Using VENNY2.1.0 software, we compared and intersected the results of the three sources (Figure 5B) and obtained 249 potential targets for QBPF on COPD treatment.

#### 3.2.2 PPI building of interaction networks and core gene screening

Utilizing the STRING database (https://cn.string-db.org/), we conducted a PPI network analysis of 249 potential targets and selected targets with a confidence score >0.9 for network construction (Figure 5C). Our results revealed the involvement of 248 nodes and 406 edges in the network. Subsequently, we imported the 176 candidate targets into Cytoscape3.8.2 to develop a visual representation of the protein interaction network. In order to identify the top 10 genes, we employed the Betweenness (Figure 5D), BottleNeck (Figure 5E), and stress (Figure 5F) algorithms in the Cytohubba plugin within Cytoscape. Finally, by intersecting the outcomes of the three algorithms, six key genes were identified, including HSP90AA1, EGFR, SIRT1, RELA, STAT3, and PTGS2.

#### 3.2.3 GO and KEGG enrichment analyses

**FIGURE 6:**
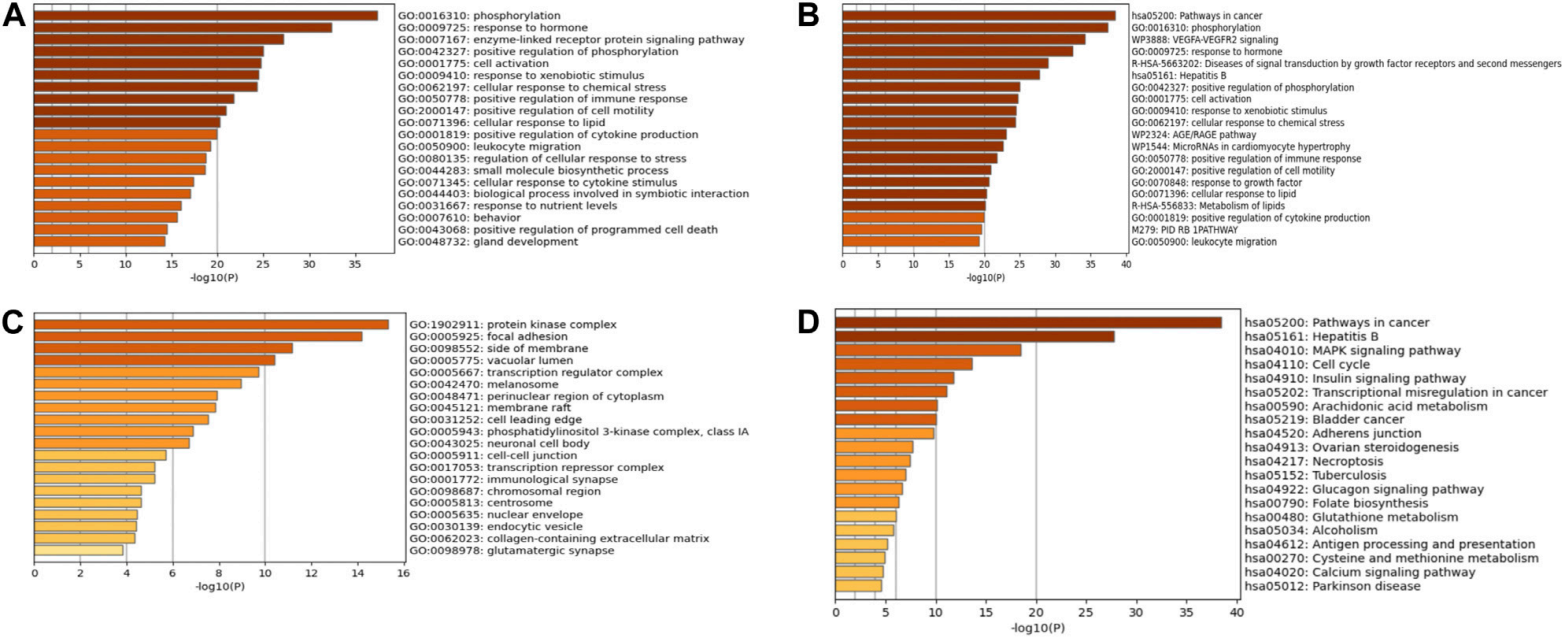
GO and KEGG enrichment analyses. **(A)** BP enrichment analysis. **(B)** CC enrichment analysis. **(C)** MF enrichment analysis. **(D)** KEGG enrichment analysis.

KEGG pathway and GO enrichment studies were conducted on 176 key targets using Metascape (https://metascape.org/) platform. The GO enrichment analysis included biological processes (BP), molecular functions (MF), and cellular components (CC). The highest 20 outcomes from the GO enrichment study were illustrated in Figures 6A–C. Simultaneously, the top 20 pathways enriched by KEGG were presented in Figure 6D.

#### 3.2.4 Molecular docking

**FIGURE 7:**
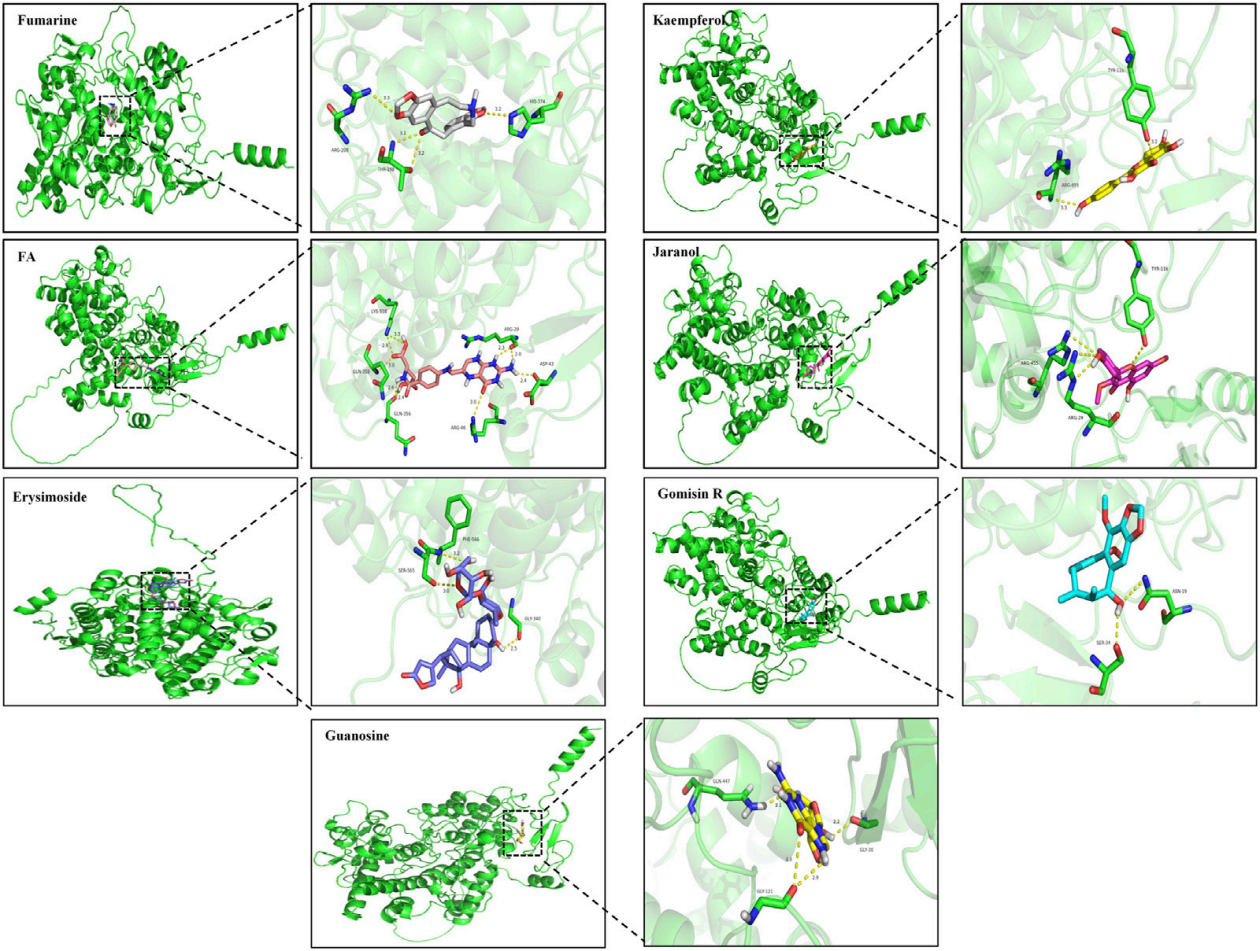
Molecular docking diagram of compounds Fumarine, Kaempferol, FA, Jaranol, Erysimoside, Gomisin R and Guanosine with PTGS2.

According to the screening results of network pharmacology, we identified 25 effective compounds that can target PTGS2. The docking results showed that 18 compounds had strong binding activity to PTGS2 (Supplementary Material S3). Therefore, we selected the top 7 compounds with the least binding activity to carry out molecular docking with PTGS2, as shown in Figure 7. Among them, Fumarine has the strongest binding to PTGS2 (−9.4 kcal/mol), followed by kaempferol (−9 kcal/mol).

### 3.3 The combined analysis of network pharmacology and metabolomics

Our metabolomics analysis revealed that 16 biomarkers were identified compared to the model group upon QBPF treatment. Out of these, six biomarkers were upregulated, while ten biomarkers were downregulated. Pathway enrichment analysis indicated that these biomarkers were mainly involved in glutathione metabolism. In addition, our network pharmacology research also showed that the KEGG enrichment analysis of the predicted QBPF targets indicated its potential to regulate glutathione metabolism. The collective findings from both analyses suggested that glutathione metabolism was the core pathway for QBPF to treat COPD.

**FIGURE 8:**
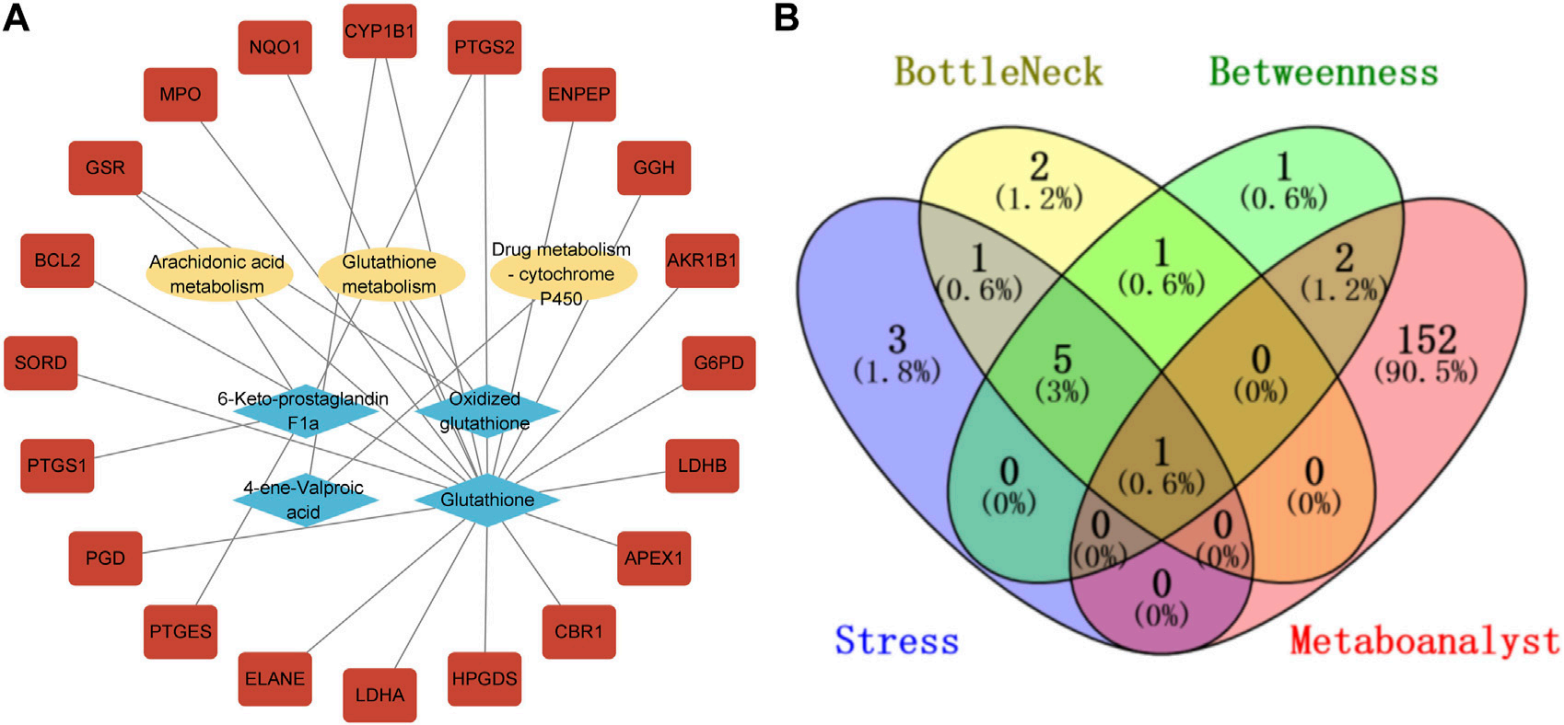
**(A)** Compound-reaction-gene networks of the key metabolites and targets. The yellow ellipsoids are differential reactions. The blue prismatics are differential metabolites. The red rectangles are differential genes. **(B)** The venny diagram of screening PTGS2.

In addition, we used the Network Analysis function in MetaboAnalyst 5.0 to conduct association prediction analysis on the callback biomarkers, and we obtained 155 predicted targets. The results showed that these recalled biomarkers were mainly clustered into three networks (Supplementary Material S4). Subsequently, a target-metabolite-metabolic pathway PPI network was established using Cytoscape, as depicted in Figure 8A. Furthermore, employing MetaboAnalyst 5.0 to predict the relevant genes of biomarkers, intersection analysis was conducted on the key genes screened by cytohubba, and PTGS2 was identified as the most critical gene regulated by QBPF (Figure 8B).

### 3.4 Animal experiments

#### 3.4.1 Lung function

**FIGURE 9:**
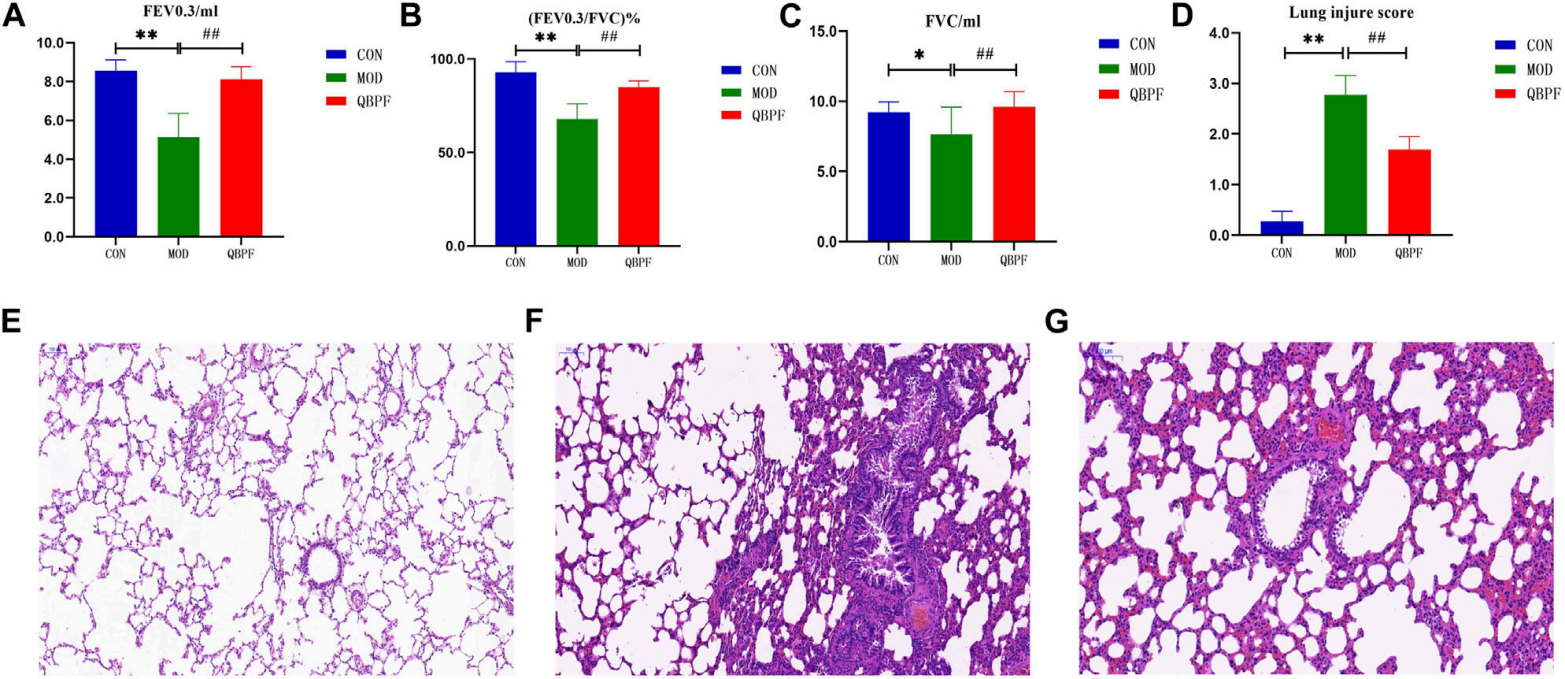
Lung function results and lung pathological features of COPD rats. **(A–C)** Pulmonary function showed FEV 0.3, FVC and (FEV 0.3/FVC) % of all groups. **(D)** Lung injure score. **(E–G)** The lung pathological features of all groups (scale bar = 200 μm). [There was difference between CON and MOD (*p < 0.05, **p < 0.01). There was significant difference between MOD and QBPF (##*p* < 0.01)].

The main feature of COPD is airway obstruction, a characteristic feature in pulmonary function test. Compared with the normal group, FEV0.3/mL, (FEV0.3/FVC)%, and FVC/mL of rats significantly (*p* < 0.05) decreased in the model group, indicated that after QBPF treatment, their pulmonary function significantly improved (Figures 9A–C).

#### 3.4.2 H&E staining

The alveolar structure of rats in the normal group showed no obvious damage (Figure 9E). The endothelial cells were normally arranged, and there were a few inflammatory cells in each layer of the tube wall. In the model group, we observed the destruction and fusion of alveolar structure, exudate in the lumen, inflammatory cell infiltration in the tube wall, and stenosis of the lumen (Figure 9F). Pulmonary lesions improved in the treatment group. Alveolar damage was alleviated and a few inflammatory cells were found in each layer of the tube wall (Figure 9G). And, We made a scoring chart for the damage degree of the rat lung tissue to visually evaluate the lung damage of the three groups of rats (Figure 9D) (FU Juan et al., 2017).

#### 3.4.3 Transmission electron microscopy indicates

**FIGURE 10:**
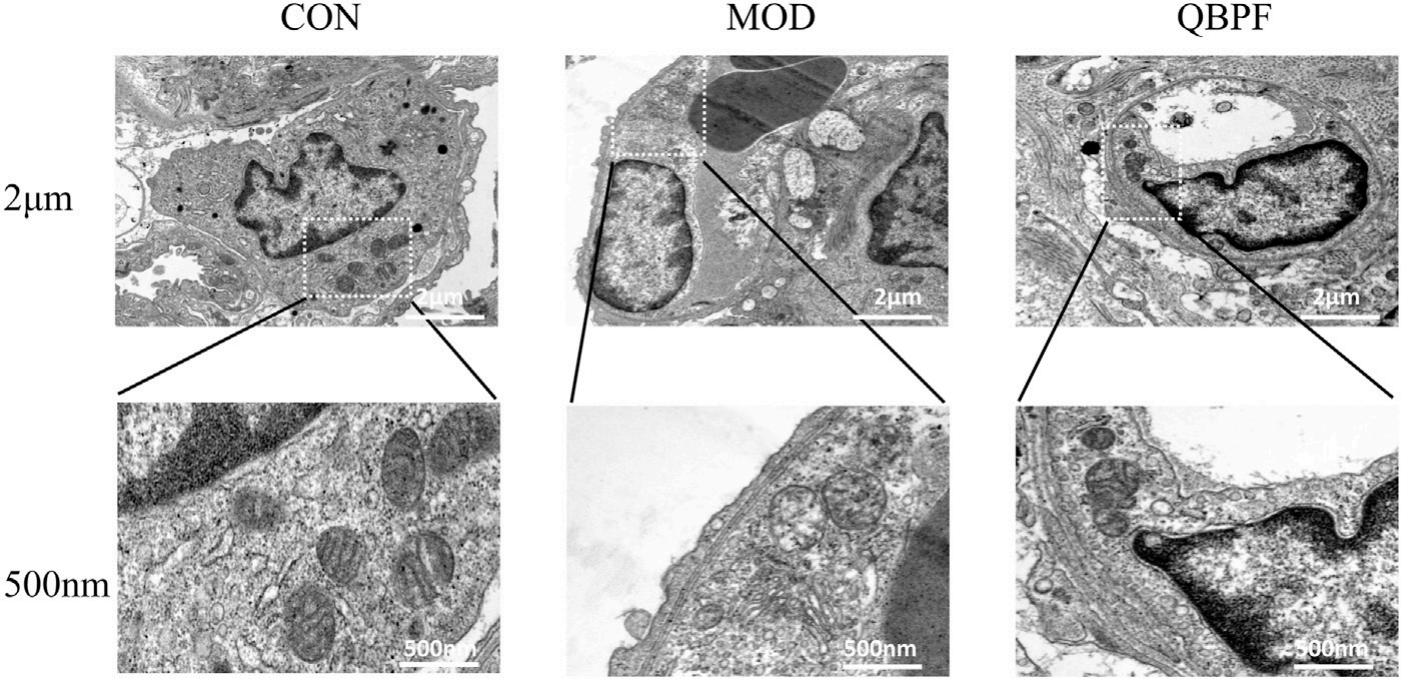
Representative TEM images of lung tissues in the different groups showed mitochondrial morphology. The model group showed the mitochondrial cristae were obviously broken and disappeared, and the mitochondria were swollen. This phenomenon improved after treatment with QBPF.

As an emerging cell death method, ferroptosis has unique pathological changes. Mitochondria serve as the primary site of action for iron, and iron overload can lead to dysfunction and structural changes. Mitochondria of ferroptosis cells generally have mitochondrial cristae fragmentation or even disappearance and mitochondrial swelling. Our electron microscopy results showed that mitochondria in the control group were granular, normal in shape and size, and mitochondrial cristae were clearly visible. In the model group, the mitochondrial cristae were obviously broken and disappeared, and the mitochondria were swollen. This phenomenon improved after treatment with QBPF (Figure 10).

#### 3.4.4 The verification of key target and ferroptosis

**FIGURE 11:**
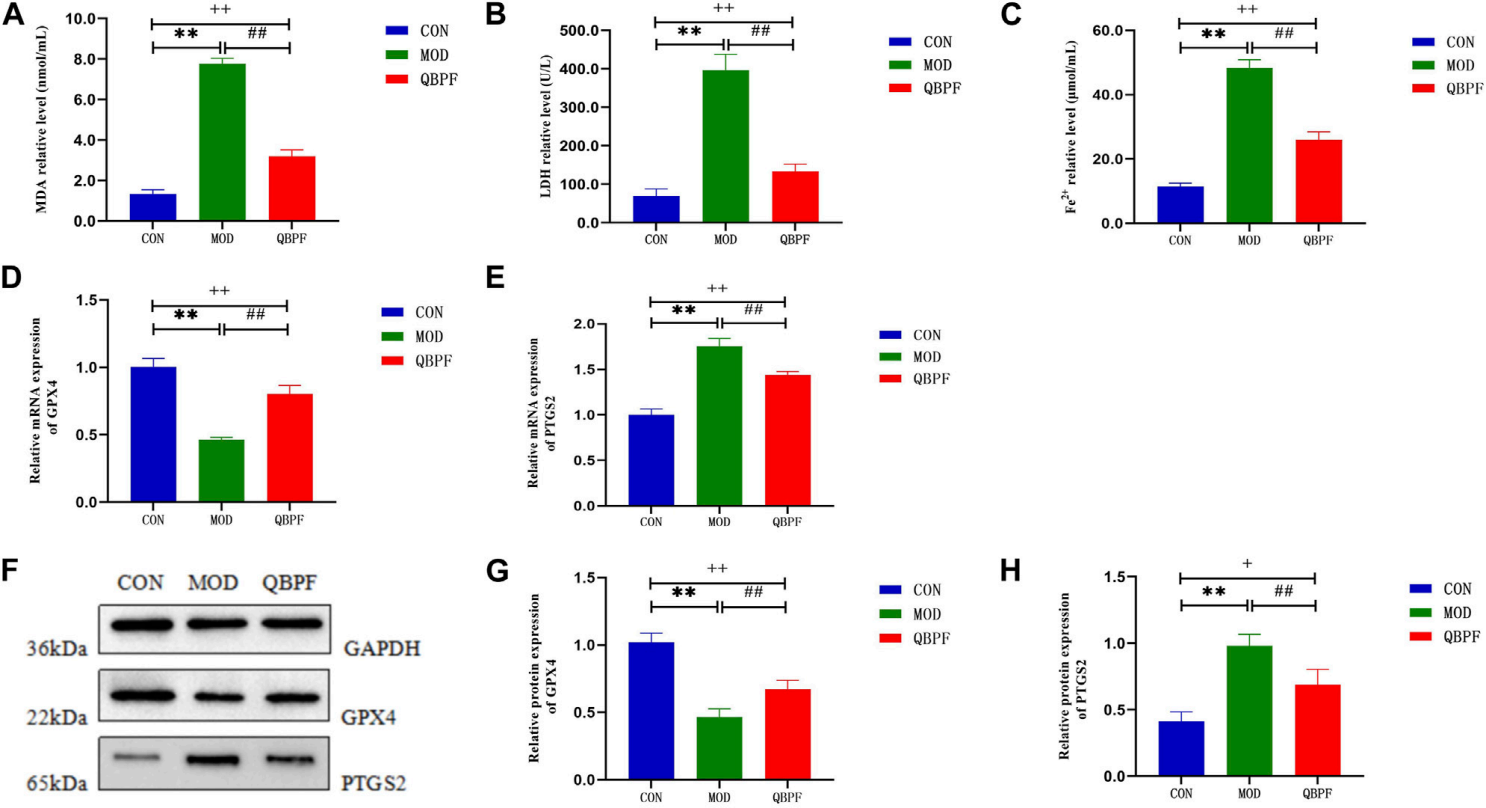
Verification of key target and ferroptosis. **(A–C)** Compared with the normal group, the content of MDA, LDH and Fe^2+^ was increased in the model group and returned after treatment with QBPF. **(D, E)** Expression of GPX4 and PTGS2 at the mRNA level by qRT-PCR. **(F–H)** Expression of GPX4 and PTGS2 at the protein level by WB [There was significant difference between CON and MOD (** *p* < 0.01). There was significant difference between MOD and QBPF (^**##**^ *p* < 0.01). There was difference between CON and QBPF (^**+**^ *p* < 0.05, ^**++**^ *p* < 0.01)].

To determine whether ferroptosis occurs in COPD and whether QBPF can ameliorate ferroptosis. We assessed indicators of ferroptosis, including the lipid peroxidation product malondialdehyde MDA, lactate dehydrogenase LDH, Fe^2+^ and glutathione peroxidase 4 (GPX4). The results showed that the contents of MDA, LDH and Fe^2+^ in lung tissue in COPD model group increased, and the levels of these indexes decreased after QBPF treatment (Figures 11A–C). The results of WB and PCR showed that the expression of GPX4 in the lung tissue of COPD rats was decreased, and it was increased after QBPF treatment (Figures 11D, F, G). The above results indicated that ferroptosis was involved in the pathogenesis of COPD, and QBPF could inhibit the ferroptosis of COPD. In addition, WB and PCR showed that the expression of PTGS2 was increased in COPD rats and decreased after QBPF treatment, which was the same as the experimental results of other researchers (Figure E–F and H).

## 4 Discussion

COPD, a serious and incurable chronic lung disease, imposes significant personal and societal burdens. Its many complications, such as pulmonary hypertension, osteoporosis, and diabetes, present challenges to its management (Adas-Okuma et al., 2019; Blanco et al., 2020; Cas et al., 2020). Currently, palliative care remains the primary approach to COPD treatment, as no highly effective remedy exists. Researchers have explored TCM due to its superior therapeutic effects and lower drug toxicity. TCM compound prescription, consisting of various Chinese herbal medicines, possesses multi-target characteristics for treating diseases. As an effective TCM compound preparation for the treatment of COPD, QBPF has unique advantages in clinical treatment. Therefore, exploring the potential multi-target mechanism of QBPF is the focus in this study.

Due to the complex compositions of Chinese herbal medicine, it may not be feasible to examine them thoroughly using a single metabolomics technology. Therefore, we have combined metabolomics and network pharmacology to analyze the therapeutic mechanism of QBPF on COPD. Our metabolomics findings indicated that 96 differential metabolites were altered in COPD rats, of which 47 had VIP >2 and were identified as biomarkers. Pathway enrichment showed that COPD was mainly associated with glycerophospholipid metabolism, sphingolipid metabolism, and glutathione metabolism abnormalities.

After treatment with QBPF, 16 biomarkers, such as glutathione, oxidized glutathione, 11-cis-retinol, d-maltose, and S-lactoylglutathione and so on, were called back. The enrichment results of metabolic pathways showed that the main effect of QBPF for COPD therapy was modulated by glutathione metabolism. Glutathione (GSH), an abundant tripeptide molecule, protects cells from oxidative stress-induced cell damage and lipid peroxidation-induced ferroptosis (Averill-Bates, 2023). Under the GPX4 catalysis, GSH reduces hydrogen peroxide and lipid peroxide, consequently oxidizing itself into oxidized glutathione (GSSG), thereby protecting cells against lipid peroxide damage (Rochette et al., 2022). The System Xc-/GSH/GPX4 pathway, with GSH at its core, is considered the crucial axis in ferroptosis (Li et al., 2022). In this study, the GSH content in COPD rats was significantly reduced. But, its content increased again after the treatment of QBPF, which is consistent with the findings of other studies (Tang et al., 2021). Therefore, QBPF may treat COPD by increasing GSH content to resist lipid peroxidation damage.

Unlike other experimental results, our study revealed a decrease in GSSG in the model group, which is slightly reversed after QBPF treatment. However, the ratio of GSH to GSSG in our results showed a decrease in the model group compared to the normal group. Subsequently, the ratio increased after QBPF treatment, which is consistent with the results of other researchers (Zhang et al., 2020) (Supplementary Material S5). Therefore, we speculate that the abnormal performance of GSSG may be linked to the related enzyme activity.

Our network pharmacology study identified 81 active QBPF components and its 176 treatment targets for COPD. Furthermore, using three cytohubba algorithms, HSP90AA1, EGFR, SIRT1, RELA, STAT3, and PTGS2 were identified as hub genes. The KEGG results indicated that these predicted targets were also involved in rno00480: Glutathione metabolism, which corresponded with our metabolomics results, and proved that QBPF treated COPD by regulating Glutathione metabolism.

In order to further investigate the efficacy of QBPF in treating COPD through glutathione metabolism, we examined the intersection of the biomarker-related genes obtained in the metabolomics section and the eight core genes identified in network pharmacology. Finally, we identified PTGS2 as the core target of QBPF that regulated glutathione metabolism. It was determined by molecular docking that active ingredients, such as kaempferol, exhibited good docking capabilities with PTGS2.

The gene encoding PTGS2, also known as cyclooxygenase 2 (COX-2), is located on chromosome 1 (Mongini et al., 2014). PTGS2 is a rate-limiting enzyme in the synthesis of prostaglandins (PGs), which catalyzes the metabolism of arachidonic acid (AA) into various PG products including the precursor prostaglandin H2 (PGH2) and leukotrienes (LTs) (Wang et al., 2023). PTGS2 plays an important role in lung diseases. In acute lung injury, the impact of NLRP3 inflammasome could be reduced by inhibiting the expression of PTGS2 (Yang et al., 2020). Similarly, PTGS2 is involved in the inflammation of COPD which is a chronic inflammatory disease. Studies showed that CS promoted the expression of PTGS2, which in turn induces inflammation in COPD (Park and Christman, 2006). As the essential transcription of PTGS2, ROS can promote its expression (Dranitsina et al., 2016). While GSH can effectively inhibit ROS generation in COPD patients, thereby alleviating oxidative stress and ferroptosis (Zhang et al., 2021; Wang et al., 2022).

Accumulating evidence indicated that PTGS2 is also a ferroptosis marker (Liu et al., 2022). Studies showed that CS can induce ferroptosis in epithelial cells of COPD rats, aggravating inflammatory damage in COPD (Tang et al., 2021). Inhibition of PTGS2 can reduce the occurrence of ferroptosis in COPD bronchial epithelial cells, thereby improving airway remodeling (Liu et al., 2023). Therefore, we hypothesized that QBPF may mediate PTGS2 to regulate ferroptosis and exert anti-inflammatory effects.

In order to verify whether QBPF could regulate the ferroptosis in COPD, we constructed a COPD rat model and detected the related indicators of ferroptosis. The results of biochemical detection showed that the content of MDA, LDH and Fe^2+^ in the lung tissue of COPD rats increased significantly, while the content of MDA, LDH and Fe^2+^ decreased after QBPF treatment. WB and PCR showed that QBPF could promote the expression of ferroptosis biomarker GPX4 and reduce the expression of PTGS2. The above results indicated that there was ferroptosis in the lung tissue of COPD rats, and the treatment of QBPF could inhibit the occurrence of ferroptosis in COPD. However, the specific regulatory effect of QBPF on COPD still needs our follow-up experimental verification.

## 5 Conclusion

In our study, the metabolomics component identified abnormal changes in glutathione metabolism, sphingolipid metabolism, and glycerophospholipid metabolism in COPD rats. QBPF was able to treat COPD by regulating glutathione, the core metabolite, metabolism. The prediction results of network pharmacology also confirmed that glutathione metabolism was an effective way of QBPF treatment. Combining the results of metabolomics and network pharmacology, we identified PTGS2 as the core target of QBPF for treating COPD by regulating glutathione metabolism. Similarly, our molecular docking results indicated that Fumarine, Kaempferol, FA, Jaranol, Erysimoside, Gomisin R and Guanosine were well-docked with PTGS2. Finally, we predict that QBPF may inhibit COPD ferroptosis by regulating PTGS2 and glutathione. Further follow-up experiments will verify the exact mechanism.

## Data Availability

The original contributions presented in the study are included in the article/Supplementary Material, further inquiries can be directed to the corresponding author.
