# Supplementary material for: Integration of metabolomics and network pharmacology to reveal the protective mechanism underlying Qibai Pingfei capsule on chronic obstructive pulmonary disease
Source: Front Pharmacol. 2023 Oct 18;14:1258138. doi: 10.3389/fphar.2023.1258138 (PMC10618342; doi:10.3389/fphar.2023.1258138)
Supplement: Supplementary file 1 [file DataSheet1.ZIP › Supplementary Material 1.docx]

| Pathway Name | p | Impact |
| --- | --- | --- |
| [Glycerophospholipid metabolism](https://www.metaboanalyst.ca/MetaboAnalyst/Secure/pathway/PathResultView.xhtml) | 0.0067423 | 0.0886 |
| [Sphingolipid metabolism](https://www.metaboanalyst.ca/MetaboAnalyst/Secure/pathway/PathResultView.xhtml) | 0.022128 | 0.05882 |
| [Glutathione metabolism](https://www.metaboanalyst.ca/MetaboAnalyst/Secure/pathway/PathResultView.xhtml) | 0.03803 | 0.28294 |
| [Amino sugar and nucleotide sugar metabolism](https://www.metaboanalyst.ca/MetaboAnalyst/Secure/pathway/PathResultView.xhtml) | 0.063152 | 0.06079 |
| [Taurine and hypotaurine metabolism](https://www.metaboanalyst.ca/MetaboAnalyst/Secure/pathway/PathResultView.xhtml) | 0.086845 | 0.42857 |
| [Nicotinate and nicotinamide metabolism](https://www.metaboanalyst.ca/MetaboAnalyst/Secure/pathway/PathResultView.xhtml) | 0.15696 | 0.1943 |
| [Glycerolipid metabolism](https://www.metaboanalyst.ca/MetaboAnalyst/Secure/pathway/PathResultView.xhtml) | 0.16655 | 0.01402 |
| [Retinol metabolism](https://www.metaboanalyst.ca/MetaboAnalyst/Secure/pathway/PathResultView.xhtml) | 0.16655 | 0.12575 |
| [Starch and sucrose metabolism](https://www.metaboanalyst.ca/MetaboAnalyst/Secure/pathway/PathResultView.xhtml) | 0.18543 | 0.07306 |
| [Pentose phosphate pathway](https://www.metaboanalyst.ca/MetaboAnalyst/Secure/pathway/PathResultView.xhtml) | 0.213 | 0.03232 |
| [Pyruvate metabolism](https://www.metaboanalyst.ca/MetaboAnalyst/Secure/pathway/PathResultView.xhtml) | 0.22199 | 0.0591 |
| [Galactose metabolism](https://www.metaboanalyst.ca/MetaboAnalyst/Secure/pathway/PathResultView.xhtml) | 0.26551 | 0.0338 |
| [Biosynthesis of unsaturated fatty acids](https://www.metaboanalyst.ca/MetaboAnalyst/Secure/pathway/PathResultView.xhtml) | 0.33814 | 0.0 |
| [Primary bile acid biosynthesis](https://www.metaboanalyst.ca/MetaboAnalyst/Secure/pathway/PathResultView.xhtml) | 0.41089 | 0.02239 |
| [Purine metabolism](https://www.metaboanalyst.ca/MetaboAnalyst/Secure/pathway/PathResultView.xhtml) | 0.53441 | 0.01945 |
