# Supplementary material for: Integration of metabolomics and network pharmacology to reveal the protective mechanism underlying Qibai Pingfei capsule on chronic obstructive pulmonary disease
Source: Front Pharmacol. 2023 Oct 18;14:1258138. doi: 10.3389/fphar.2023.1258138 (PMC10618342; doi:10.3389/fphar.2023.1258138)
Supplement: Supplementary file 1 [file DataSheet1.ZIP › Modified—Supplementary Material 3.docx]

| Ligand | Binding Affinity |
| --- | --- |
| Mairin | -7.9 |
| Jaranol | -8.4 |
| 5'-hydroxyiso-muronulatol-2',5'-di-O-glucoside | -8.2 |
| 9,10-dimethoxypterocarpan-3-O-β-D-glucoside | -7.5 |
| kaempferol | -9 |
| FA | -8.6 |
| isomucronulatol-7,2'-di-O-glucosiole | -8 |
| Stigmasterol | -7.1 |
| (Z)-3-(4-hydroxy-3-methoxy-phenyl)-N-[2-(4-hydroxyphenyl)ethyl]acrylamide | -7.5 |
| Fumarine | -9.4 |
| Mandenol | -4.5 |
| senkyunone | -5.8 |
| 11,14-eicosadienoic acid | -4.5 |
| Diop | -6.9 |
| erysimoside | -8.3 |
| Cynotoxin | -7.9 |
| Dihomolinolenic acid | -5.8 |
| Chrysanthemaxanthin | -5.8 |
| Dianthramine | -8.2 |
| Panaxadiol | -8.1 |
| Angeloylgomisin 0 | -7.7 |
| Schizandrer B | -7.1 |
| Gomisin R | -8.3 |
| Uracil | -5.5 |
| Guanosine | -8.3 |
